# Nutritional Enteric Failure: Neglected Tropical Diseases and Childhood Stunting

**DOI:** 10.1371/journal.pntd.0004523

**Published:** 2016-04-28

**Authors:** Kirkby D. Tickell, Judd L. Walson

**Affiliations:** 1 Department of Global Health, University of Washington, Seattle, Washington, United States of America; 2 Departments of Global Health, Pediatrics, Medicine, and Epidemiology, University of Washington, Seattle, Washington, United States of America; University of Texas Medical Branch, UNITED STATES

Chronic malnutrition, defined by linear growth failure, or stunting, affects over 165 million children globally [1]. In many areas of the world with a high prevalence of stunting, children experience frequent and recurrent exposure to pathogens, including neglected tropical diseases (NTDs). These infections appear to have detrimental effects on linear growth [2–6], but interventions to promote linear growth have demonstrated limited benefit. Difficulty in establishing effective growth-promoting interventions is not unique to NTDs; even the optimal delivery of all interventions known to improve nutritional status is estimated to be able to reverse less than a quarter of all stunting [7]. The failure to identify effective interventions to reverse stunting offers the opportunity to develop a new conceptual model of chronic malnutrition that furthers our understanding of the mechanism linking pathogen and environmental exposures to linear growth failure. Such a conceptual model may guide the identification of new targets for intervention to reduce the substantial morbidity and mortality associated with chronic malnutrition [1].

The current definition of chronic malnutrition is based on anthropometric indicators suggesting previous or ongoing stunting. However, stunting and its associated cognitive and immunologic sequelae represent the end stage of a complex series of pathophysiologic events. Fundamentally, it is the failure of the enteric system to meet the metabolic demands of the growing and developing child, as a direct result of either inadequate dietary intake, poor absorption of energy and nutrients, chronic inflammation, or interactions between these etiologies [2]. The failure of the gut as an organ system to meet the metabolic and immunological demands of the growing child can be classified as “nutritional enteric failure,” characterized by linear growth failure, decreased cognitive development, and susceptibility to infection. All of these insults directly relate to the failure of the enteric system to meet the specific needs of tissues with high metabolic demand, including bone, the brain, and the immune system. Furthermore, the gut may also fail in its two key immune functions: firstly as a barrier to infection and secondly as an important antigen-processing organ. Reclassifying the stunting syndrome as nutritional enteric failure would shift focus away from decreased height and highlight the underlying mechanisms linking postnatal environmental influences to linear growth failure and its associated morbidity, and it would enable the identification of additional interventional targets to prevent substantial childhood morbidity and mortality.

**Fig 1:**
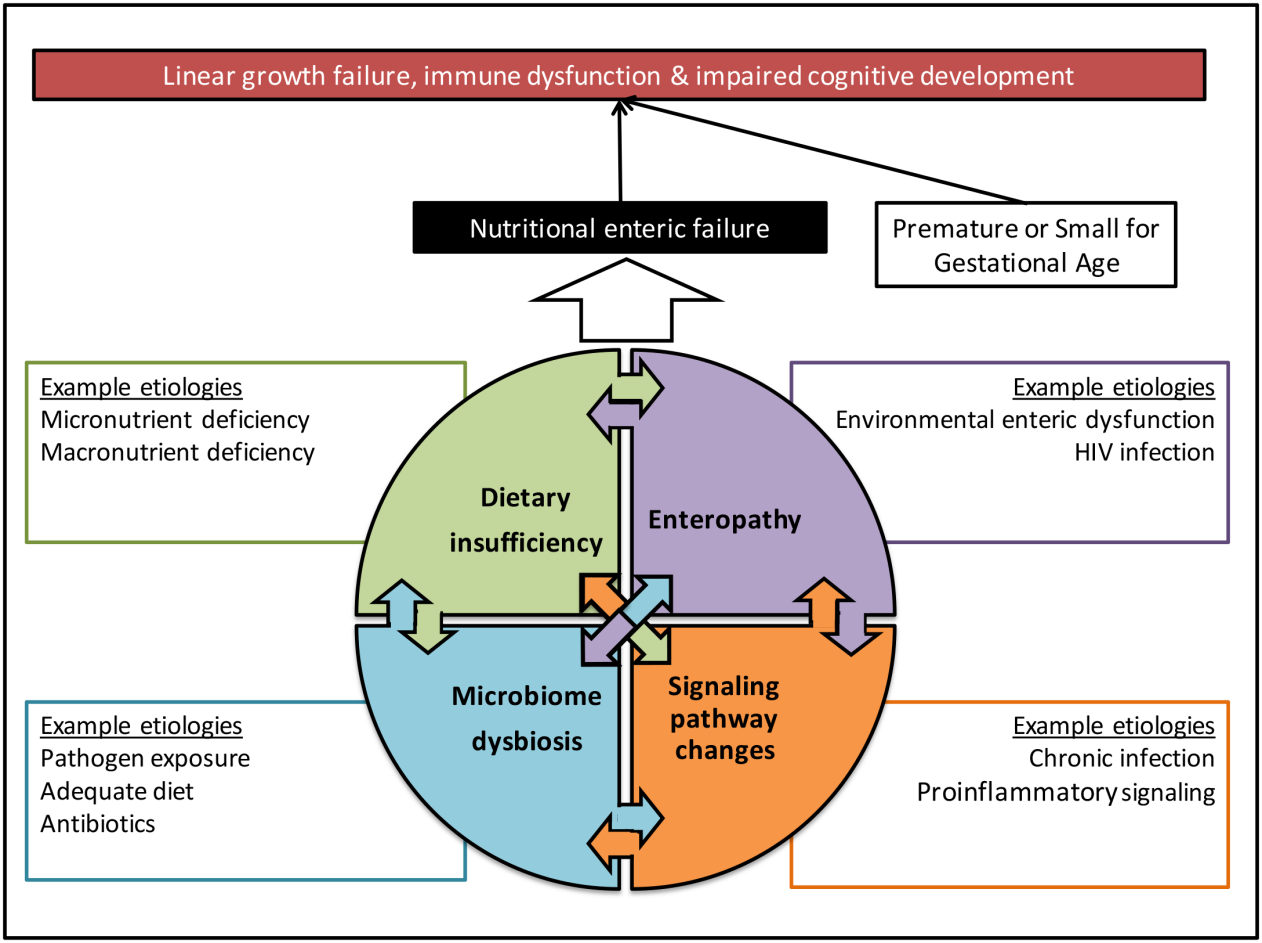
The proposed role of nutritional enteric failure in linking diverse and overlapping etiologies to its key signs of vulnerability to infection, developmental delay, and linear growth failure.

Organ failure is defined as a condition in which a system cannot maintain normal homeostasis, resulting from either insufficient supply or inadequate excretion of a substrate in relation to a patient’s metabolic requirements. For example, heart failure is defined as a state in which the cardiac output is unable to meet the body’s circulatory demands without external support [8]. In a child with chronic malnutrition, the enteric system fails to meet the metabolic demands of normal growth and development. As in heart failure, this may result from a lack of substrate (caloric or nutrient), maladaptive structural or pathophysiological changes (enteropathy), or increased metabolic demands that outpace the ability of the enteric system to meet these energy requirements (chronic infection or inflammation). However, like other organ failure syndromes, nutritional enteric failure rarely has a single cause and is more accurately described as a final common pathway of multiple interconnected pathologies (Fig 1).

Three interacting pathways define nutritional enteric failure: environmental enteric dysfunction (EED), enteric microbiome dysbiosis, and systemic inflammation. EED is a syndrome of decreased intestinal barrier integrity, and villus blunting, resulting from persistent or repeated exposure to pathogens and contaminants in the environment, appears to be an important contributing factor to linear growth failure [9,10]. The alterations to the gut architecture caused by EED decrease the absorption of nutrients and concurrently increase inflammation, essentially reducing supply while increasing metabolic demand [9–13]. However, while enteric inflammation is highly prevalent in children living in lower-income countries, many of these children do not suffer immediate, clinically apparent complications of nutritional enteric failure, such as growth failure or cognitive delay. These children may suffer subtler manifestations, such as vaccine failure or increased incidence of infection, and could be spared the more obvious sequelae because nutritional enteric failure, similar to other organ failures, may be preceded by a period of compensated dysfunction, in which a child with enteric inflammation utilizes stored nutrients and fats or has excess functional capacity within the gut to compensate. However, once the excess capacity or nutrient reserves have been depleted, the body decompensates and linear growth failure begins. Targeting children at high risk before decompensated enteric failure occurs may offer the opportunity to improve the effectiveness of available interventions.

The enteric microbiome also appears to play a key role in maintaining homeostasis through vitamin production, mineral absorption, and regulating immune function and metabolic activity [14–16]. Chronically malnourished children have an immature microbiome, and these abnormalities appear associated with linear growth failure [17]. Interestingly, transplant of the microbiome from a malnourished child to a mouse model appears to result in a similar failure of the enteric system to support growth, suggesting that there are transferrable elements of the microbiome that result in growth failure, particularly when combined with certain diets [15,18].

Finally, alterations in signaling pathways as a result of systemic inflammation can lead to a highly catabolic state with increased energy demands and reduced appetite [13,19]. Cytokines, including interferon γ and interleukin-6, appear to reduce micronutrient uptake and increase bone resorption [19,20]. Such a state of immune activation also appears to dramatically impact host immune function [2,13,20]. In fact, over 40% of all childhood diarrheal and pneumonia deaths are associated with stunting [21]. These chronically malnourished children also exhibit decreased oral vaccination efficacy. For example, rotavirus vaccine has a demonstrated efficacy as high as 98% in high-income countries but has an efficacy of only 58% in Nicaragua and 46% in Bangladesh, where stunting is highly prevalent [13,22–25].

When we view available interventions through the lens of nutritional enteric failure, our inability to meaningfully promote linear growth is unsurprising. Stunting is likely to be the late sign of a prolonged disease process, even in infants, as it takes many months to cross multiple centile lines between their natural height and the stunting cutoff. Targeting intervention packages at stunted children is likely too late in the disease process to achieve meaningful catch-up growth. However, identifying children with the underlying causes of nutritional enteric failure and intervening earlier in the disease trajectory may offer opportunity to reverse the underlying pathology, reduce morbidity, and improve growth and cognition.
